# Application of information-intelligence technologies in pharmacy intravenous admixture services in a Chinese third-class a hospital

**DOI:** 10.1186/s12913-022-08580-4

**Published:** 2022-10-07

**Authors:** Xu Wang, Ming Gu, Xueqin Gao, Xiang Xiong, Nanxi Wang, Qiuqi Li, Miaomiao Ge, Miao Luo, Yu Zhang, Xiaoli Hua, Chen Shi

**Affiliations:** 1 Department of Pharmacy, Union Hospital, Tongji Medical College, Huazhong University of Science & Technology (HUST), Wuhan, People’s Republic of China; 2 Hubei Province Clinical Research Center for Precision Medicine for Critical Illness, Wuhan, People’s Republic of China

**Keywords:** PIVAS, Information-intelligence technologies, Prescription review, Increase efficiency, Reduce errors

## Abstract

**Background:**

Pharmacy intravenous admixture service (PIVAS) center has emerged as an important department of hospital as it can improve occupational protection and ensure the safety and effectiveness of intravenous infusions. However, medication errors were considered to be a significant challenge in PIVAS, so information-intelligence technologies were introduced to optimize the management of PIVAS. Our article summarized the application of information-intelligence technologies in PIVAS of a large third-class A hospital in China, and provided an example for PIVAS in other hospitals at home and abroad.

**Methods:**

Prescription-reviewing rules containing intravenous medications and infusion solution guideline were recorded in the database of prescription-cheking system. Drugs information were recorded in the PIVAS management system with special identification and warning labels to reduce intravenous infusion errors. Automatic labeling device was used to label the infusion bags, and the quality control program database of intelligent compounding robot for cytotoxic drugs was established ingeniously. Automatic sorting devices were applied for the third batch of finished infusion admixtures, and intelligent logistics robots were used to transport the infusion to the ward.

**Results:**

After establishing and implementing of prescription-reviewing rules in the prescription-cheking system database, the number of prescriptions checked by pharmacists increased from 18 to 43 per minute. The success rate of intervention with irrational medical orders increased from 85.89% to 99.06% (*P* < 0.05). By introducing various intelligent devices, automatic labeling significantly enhanced work efficiency and reduced the error rate (*P* < 0.001). Furthermore, the use of intelligent intravenous compounding robots significantly reduced the risk of errors (*P* < 0.001).

**Conclusions:**

The application of information-intelligence technologies in PIVAS can improve work efficiency and reduce error risk. However, some intelligent devices have failed to achieve the expected effect in practical use, and further improvements are needed to meet the demands of PIVAS in the future.

## Introduction

According to the “Regulations on the Administration of Pharmaceutical Affairs in Medical Institutions” issued by the Ministry of Health of China in 2002, medical institutions should establish Pharmacy Intravenous Admixture Services (PIVAS) for anticancer chemotherapeutic drugs and Total Parenteral Nutrition (TPN) based on clinical demands [1]. The requirements and standard operating procedures for PIVAS were also described in the “Regulations on the Quality Management of Pharmacy Intravenous Admixture Services” issued by the Ministry of Health in 2010. In recent years, remarkable progress has been made in the establishment of PIVAS, which has become an important part of hospital pharmacy [2]. PIVAS facilitates the centralized dispensing of drugs previously distributed in different wards thus playing an important role in the occupational safety of intravenous drugs dispensing [3]. The centralized compounding of intravenous drugs can avoid contamination of intravenous drugs to the greatest extent during the compounding process, effectively ensure the safety of patients' medication, and at the same time increase the efficiency of hospital compounding, reduce the workload of nurses in wards and the risk of drug exposure, promoting standardized management in hospitals [4]. However, PIVAS collects the intravenous drugs that were previously dispersed in various wards, and compounding them in a specific sterile environment, once a PIVAS error occurs, it will cause serious consequences, not only causing waste of drugs and affecting the medication of a certain patient., and even group errors may occur [5]. With the increasing amount of PIVAS in large hospitals, excellent management guidelines have become a necessary to condition eliminate medication errors [6].

Founded in 1866, our hospital (Union Hospital, Tongji Medical College, Huazhong University of Science and Technology) is a comprehensive public hospital directly under the National Health Commission, a “double first-class” university affiliated hospital (the First Clinical School), a national third-class and top 100 hospitals in China. In the “Chinese Hospital Rankings” released by the Institute of Hospital Management of Fudan University, comprehensive strength of disciplines ranks among the top 10 in China. Our PIVAS was established at the end of 2018 and certified by the Health Commission of Hubei Province. The PIVAS has a working area of approximately 1440 m^2^, including centralized dispensing and transport facilities for intravenous drugs, as well as serving 5,000 patients in 96 medical wards. Moreover, it is one of the largest infusion centers in Central China with a daily combined infusion volume of nearly 10,000 cases. The 112 employees working in the PIVAS include 17 pharmacists, 73 nurses, 10 logistics staffs, 6 laborers, and 6 cleaning staff. There exits 22 horizontal laminar-flow clean benches and 15 biosafety cabinets dedicated to intravenous medications preparation.

Systematic management of nosocomial infection and information-based intelligent technology of PIVAS medication management played a critical role in ensuring the safety of infusion products and reducing the burden of clinical nurses. Especially during the coronavirus disease 2019 (COVID-19) pandemic, PIVAS ensured infusion supplies reduced infusion preparation workload and enabled nurses to provide care to COVID-19 patients [7]. The pandemic made us realize the unique advantages of “zero-contact” pharmaceutical care model based on information-intelligence technology. Intelligent devices not only improve work efficiency but also prevent cross-infection during the pandemic [8].

**Fig. 1:**
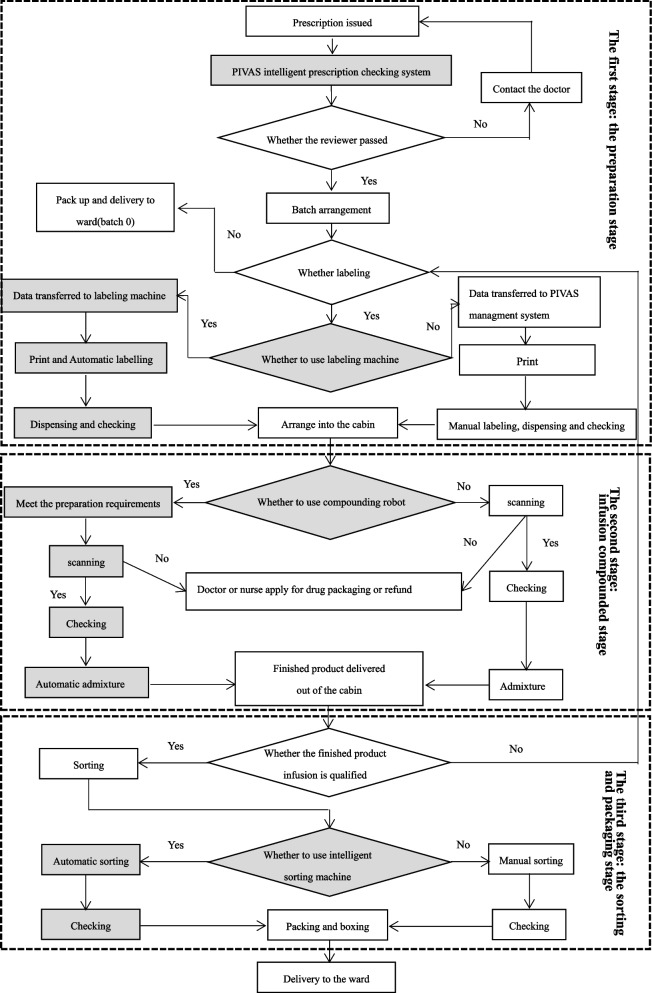
The daily workflow diagram of PIVAS. *Gray indicates optimized procedures involving intelligent technologies

In this article, we summarized our daily workflow (Fig. 1). The workflow of PIVAS in our hospital was divided into three stages: preparation, intravenous infusion compounding, product sorting and packaging. The PIVAS management system was embedded into the hospital information integration platform, and the medical orders from the electronic medical record system (EMR) were then transferred to the PIVAS management system. The medical order reviewing was followed by the input of the intravenous infusion medication order in the EMR (by the physician) containing review of intelligent prescription system and pharmacists. After accounting, batch arrangement, labeling, compounding and packaging of infusion products by Hospital Information System (HIS), they were delivered to the inpatient ward and received by nurses. Before infusion compounding, the staff would scan it. If patient's infusion needed to be packed back to the ward or refunded, the pharmacist will deal with them separately.

Before we introduced various intelligent technologies, the main workflow of PIVAS was manual. Although some intelligent approaches have been introduced, the function was not completely suitable for PIVAS, such as some rules in the prescription-checking database purchased by the hospital did not conform to PIVAS. Therefore, commercial prescription-checking database did not play its due role in warning pharmacists, resulting in wasting a lot of time on manual review. It has been reported that adverse reactions caused by toxic agents were associated with frequent exposure to chemotherapy drugs [9]. We entirely relied on manual preparation of toxic drugs for a long time, which resulted in a high risk of exposure to toxic drugs. There were 58 kinds of chemotherapy drugs in our PIVAS, with approximately 30,000 bags infusion annually. Since the same drugs were mixed uniformly according to different batches, it was important to sort the prepared infusion accurately and quickly according to the ward. Due to the large amount of infusion bags, the sorting required lots of staff to ensure timely supply [10]. The first 6000 infusion bags should be sorted by 8:15 and delivered to the wards by 8:45. Manual sorting speed should reach 134 bags per min, which meant that each worker was required to sort 430 bags on average. This process was time-consuming, labor-intensive and even led to delays in the delivery of prepared infusion products. However, manual sorting required too much manpower, bringing about increased costs, chaos in a limited space, and high possibility of errors.

At present, how to decrease the labor cost and complete the whole process of PIVAS accurately and quickly is still the main problem for large-scale PIVAS. Fortunately, the application of intelligent technology provided the possibility to solve these problems. This study aimed to introduce the experience of PIVAS informatization and intelligent application in a large third-class A hospital in China, so as to provide reference for PIVAS at home and abroad. This paper systematically summarized the management experience of PIVAS in recent three years, aiming to promote the exchange of PIVAS pharmacists in domestic and foreign hospitals, and provide a reference for the future development of PIVAS. We also provided guidelines on how to deal with large numbers of infusions, as well as how to reform the management system of intelligent equipment based on the working conditions and clinical demands.

## Methods

### Participants

Pharmacists and nurses in the PIVAS jointly participated in our study. The pharmacists were responsible for the collection of data related to the prescriptions checking, and the nurses were responsible for collecting data related to working efficiency and error incidence of intelligent equipments compare to the manual operation in infusions labeling, intravenous drug admixing and finished product sorting.

### Application of information-intelligence technologies in management of PIVAS

#### Establishment of intelligent prescription-checking system

**Fig. 2:**
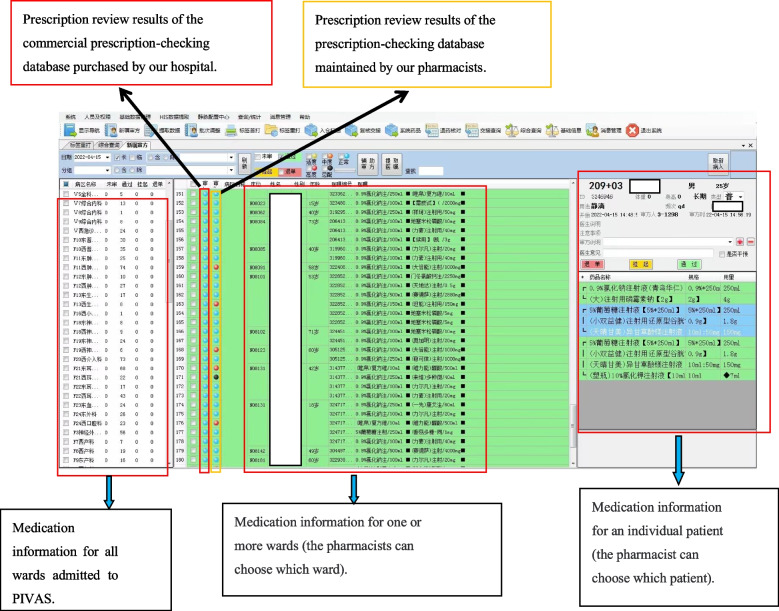
The interface of the double prescription-checking of PIVAS

Rules of prescription checking were improved and the database for prescription checking was built up for improving accuracy and efficiency of prescription audit operation, which based on the commercial prescription checking software. The two warning lights were used to reduced the risk for medications review errors (Fig. 2) [11–13]. In the system, blue light indicated correct prescriptions, red light indicated the prescription should be reviewed by a pharmacist, while black light indicated contraindications in the prescription and pharmacists should pay more attention. Pharmacists reviewed prescriptions online quickly and accurately after establishinng of the intelligent prescriptions checking system. Moreover, pharmacists can click on warnings notes to review medication instructions and related treatment guidelines in the system conveniently and quickly. Compared with prescriptions checking only by pharmacists, work burden and incidence of error in prescriptions checking were reduced and the working efficiency was also improved.

#### Application of intelligent equipments in PIVAS

In the study, automatic infusion labeling machine(KLMT-02 ALM-2020), intelligent intravenous compounding robot (WEINAS-VD160/WEINAS-PD 160), intelligent infusion bag sorting equipment (KLMF-01 SSM-2020) and intelligent logistics robot (TRV-08) were applied to infusions labeling, intravenous cytotoxic drug compounding, finished products sorting and delivering, respectively. Aim of this study was to determined the effect on reducing work burden and error incidence and improving work efficiency after intelligent equipments application in the PIVAS.

### Data collection

We collected the datas including: (1) before and after the establishment of the intelligent prescriptions check sysytem from 2019.07.01 to 2019.09.30 and from 2020.07.01 to 2020.09.30, analyze and compare the differences between the two groups of the rate of irrational prescriptions, the number of successful interventions, and the success rate of interventions were compared; (2) the datas on work efficiency and error incidence of intelligent equipments and manual operation for 60 days were collected, and the differences were also evaluated.

### Data analysis

Data was expressed as x ± s, t test or analysis of variance was used. Categorical variables were analyzed by the chi-square test. The difference was statistically significant at *P* < 0.05. Data were analyzed using SPSS 22.0 (IBM Corp., Armonk, NY, U.S.A).

## Results

### The achievements of informationization transformation of PIVAS

**Fig. 3:**
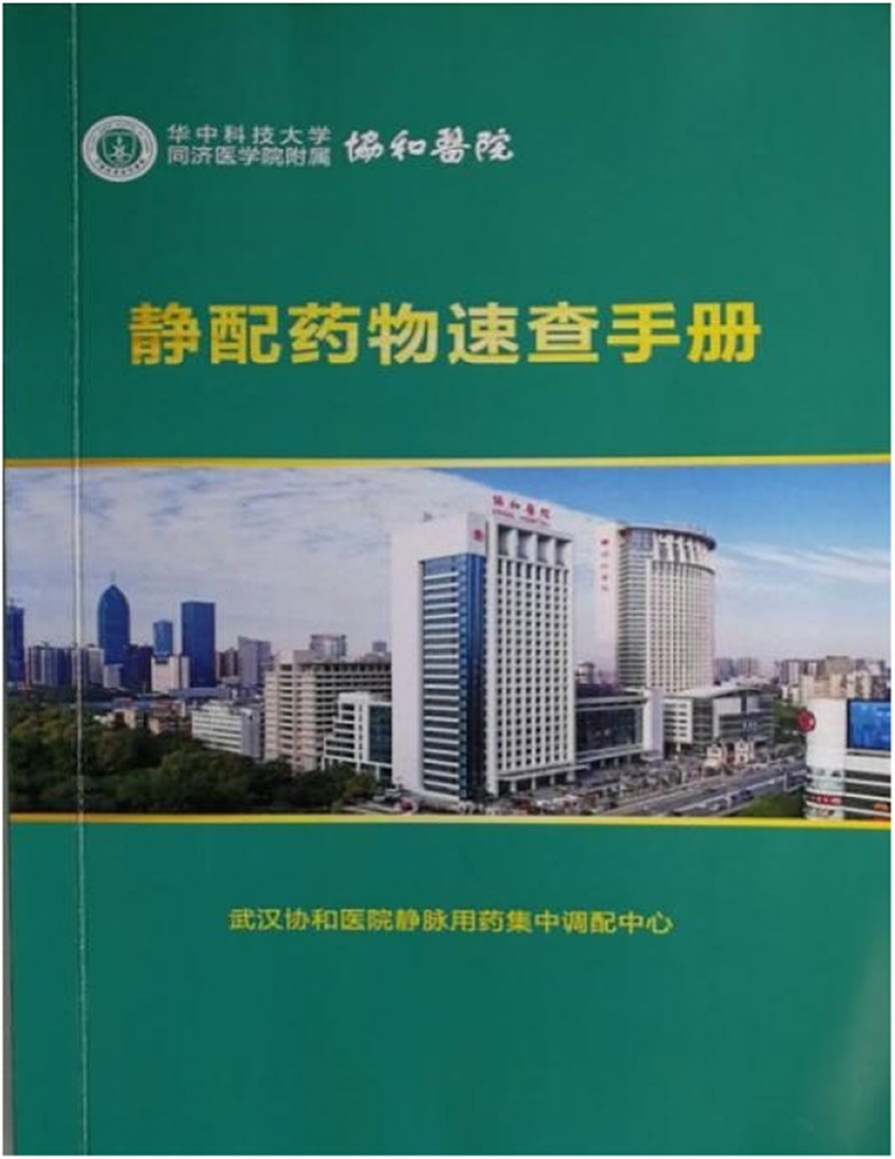
The Quick Check Manual for Intravenous medications book including guidelines for prescription reviewing

We have established guidelines for reviewing prescriptions of 368 drugs and 28 solvents (containing amino acids and lipid emulsions) by the custom-built prescription review system. The Quick Check Manual for guidelines for prescription reviewing of intravenous medications (Clinical Intravenous Drug Data) was also compiled by the pharmacists in PIVAS (Fig. 3), and provided to the medical staffs in clinical departments. The contents of the book include prescription-checking rules for intravenous drip drugs, maximum dosage rules for commonly used solvents, drug checking rules for parenteral nutrition, etc. The review guidelines referenced the commercial prescription-checking software of PIVAS purchased by our hospital,the drug instructions and the practical experience of PIVAS. In the early stage of PIVAS, 17 professional pharmacists spent six months continuously inputting the rules for reviewing prescriptions into the PIVAS commercial software .

We mainly established the following three aspects of the prescription-checking rules.

1. Established the prescription-checking rules related to the intravenous medication

**Fig. 4:**
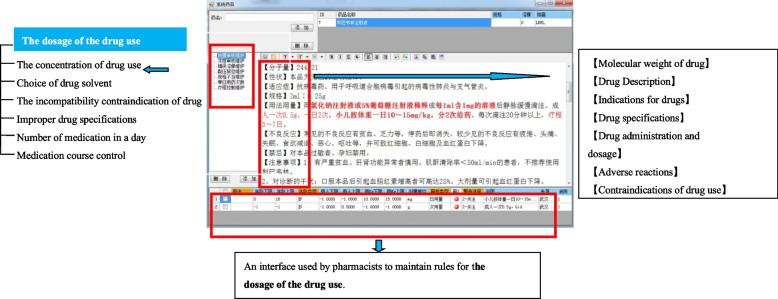
Interface for pharmacists of prescription-checking rules in PIVAS

Irrational intravenous prescriptions include wrong solvent, incompatibility, inappropriate concentration, inappropriate dosage, and wrong route of administration [14]. By establishing a database of prescription-checking irrational prescriptions could be corrected before accounting. The database were established based on drug instructions, clinical medication instructions, diagnosis and treatment guidelines, as well as the working experiences of PIVAS and clinical requirements. Examples of some prescription-checking rules are shown in Table 1. As shown in Fig. 4, the key contents were marked in red to remind pharmacists. And this interface is also the interface for pharmacists to maintain prescription checking rules. Through this interface, pharmacists can add new prescription checking rules for drugs.

2. Established the prescription-checking rules related to the infusion solution

**Table 1:** Examples of prescription-checking rules for intravenous drugs

Type	Irrational prescriptions	Prescription-checking rules and warnings
Wrong solvent	Lansoprazole for injection/30 mg + 5% GS/100 mL Carboplatin injection/0.4 g + 10% GS/250 mL Homoharringtonine injection/2 mg + 0.9% NS/100 mL	0.9% NS only 5% GS 250—00 mL only 5%or10%GS 250–500mlL
	Hepatocyte growth-promoting factor injection/120 mg + 0.9%NS/250 mL	GS only
	Dacarbazine injection/0.6 g + 0.9% NS/250 mL	5% GS 250–500 mL only
Incompatibility	Vitamin C injection/2 g + calcium aspartate injection/1.5 g Dexamethasone injection/3 mg + cefazedone sodium injection/0.5 g + 5% GS/100 mL	Incompatible Incompatible
	Vitamin C injection/2 g + riboflavin sodium phosphate injection	Affectthetherapeutic effect
Inappropriate concentration	Ribavirin injection/500 mg + 0.9% NS/250 mL 10% potassium chloride injection/10 mL + invert sugar injection/250 mL	≤ 1 mg/mL ≤ 3 mL/100 mL
	Etoposide injection/150 mg + 0.9% NS/250 mL	≤ 0.4 mg/mL
Inappropriate dosage	Propacetamolhydrochlorideinjection/4 g + 0.9% NS/100 mL Vinpocetine injection/60 mg + GS/250 mL	Adults (and children over 15 years old): 1–2 g/dose Adults: 20–30 mg/dose
	ademetionine1,4-butanedisulfonate injection/1.5g + 5% GS/250 mL	Adults: 0.5–1 g/dose
Route of administration	Mouse nerve growth factor injection/18 μg + 0.9% NS/100 mL Thymopentin injection/40 mg + 0.9% NS/100 mL	Intramuscular injection Hypodermic injection

**Fig. 5:**
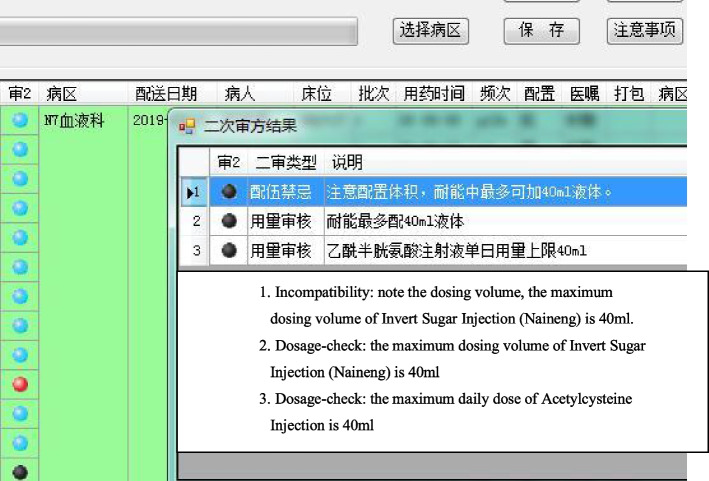
Warning message for excessive infusion volume

**Fig. 6:**
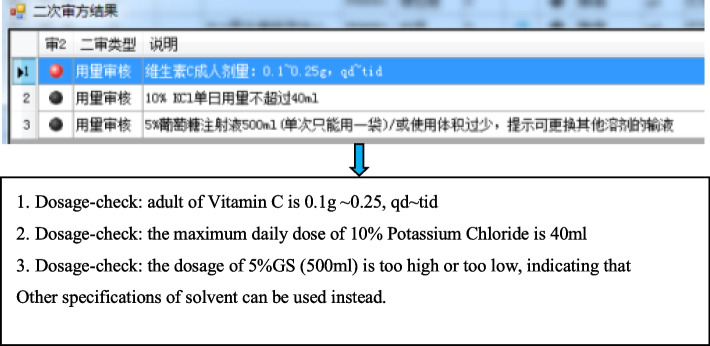
Warning of inappropriate infusion specification

Exceeding the maximum volume of the infusion bag may cause incomplete compoundin of drugs in intravenous infusions and rupture of the infusion bag during transportation, which leading to drug waste. The maximum volume of common solvents were determined by PIVAS management systems according to the working experience (Table 2). If the total volume of infusions is exceeded, a warning message will be issued (Fig. 5) to remind the pharmacist to correct the prescription immediately. The drugs with different requirements regarding the amount of solvent (Table 3) for the accuracy of drug concentration and the infusion time. The irrational prescriptions can be corrected before proceeding to the next step by appropriate warning messages (Fig. 6).

3. Established rules for batch adjustment

**Table 2:** Prescription-checking rules for infusion volume

Type of solvent (generic name)	Specifications(mL)	Maximum dosingvolume of solvent (mL)
0.9% NS/5% GS	50	20
0.9% NS/5% GS	100	30
0.9% NS/5% GS	250	60
0.9% NS/5% GS	500	100
10% GS	250	50
GNS	500	100
Fructose injection	250	50
Invert sugar injection	250	50
Invert sugar and electrolytes	500	100
Sodium acetate ringer’s injection	500	100
Multiple electrolytes	500	80
Carbohydrates and electrolytes	500	100

**Table 3:** Amount of solvent requirements

Drug	Infusion time	Solvent volume
Gemcitabine	30 min	l00–250 mL
Docetaxel	60 min	150–250 mL
Cyclophosphamide	≤ 3 h	≤ 250 mL
Lobaplatin	≤ 4 h	≤ 250 mL

The medical orders of all patients are divided into several batches, each batch is composed of several groups of medical orders, and the same batch is sent to the ward at the same time, so as to ensure that patients who have been issued intravenous medical orders can have infusion at 8:30 a.m. Prescriptions from 96 wards are automaticly divided into 0 to 7 batches based on the doctors’ instructions, drug characteristics, medication frequency, infusion volume etc., after being reviewed by pharmacists. Batches 1 to 4 were long-term prescriptions, and batches 5 to 7 are temporary prescriptions. There are many types of frequency of administration, including qd, q12h, q8h, q6h, bid, tid, etc. Due to limited staff, those drugs with multiple-dose administration need to be packaged and sent to the ward for temporary compounding, and the relevant prescriptions have been put into batch 0 according to the prescription-checking rules in advance. After batch adjustment, infusion labels were printed according to the batch sequence and automatically distributed to 12 groups via the PIVAS management system. There were two people in each group: one adding the labels while the other one checking.

**Fig. 7:**
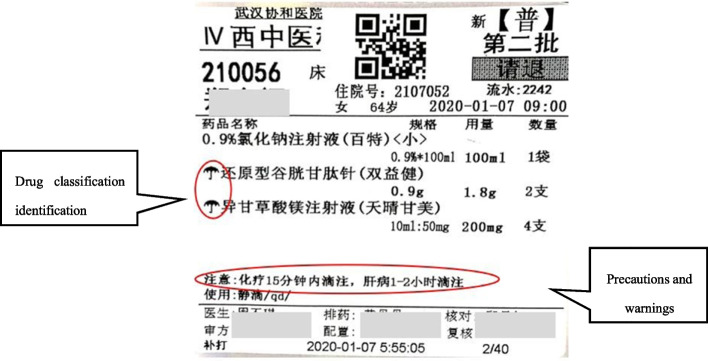
Infusion label with special markings and warnings

Furthermore, to remind nurses to check the precautions before administering intravenous infusions and minimize the mistakes, identification marks and warnings were printed on the infusion label [15]. At the same time, special precautions drug administration methods (e.g., drip speed, and administration time), storage methods (e.g., protection from light and refrigeration), and drug attributes (e.g., high-alert drugs, cytotoxic drugs) were presented as “drug precautions” on the infusion label (Fig. 7). In the infusion label, different eye-catching symbols are used for different drugs.

### Comparison of pre and post-implementation of the prescription review system

#### Improved irrational medical orders intervention

The established prescription-checking system can be also used by clinical medical staff to access the PIVAS system, identify the reasons for medical order errors and intervene irrational prescriptions as early as possible. With the intelligent prescriptions review system and the handbook of “Clinical Intravenous Drug Data” provided to clinical medical staff for reference, the intervention rate of irrational medical orders was improved.

We analyzed the rate of successful medical order intervention before and after the establishment of the rule database. Medical orders between 2019.07.01 and 2019.09.30 (Group 1) and between 2020.07.01 and 2020.09.30 (Group 2) were reviewed. The number of irrational medical orders, the rate of irrational prescriptions, the number of successful interventions, and the success rate of interventions were compared. From 2019.07.01 to 2019.09.30, there were 709,040 medical orders, of which 16,488 were irrational medical orders. The rate of irrational medical orders was 2.33%, and the success rate of intervention was 85.89%. By comparison, from 2020.07.01 to 2020.09.30, there were 687,656 medical orders, of which 11,446 were irrational medical orders. The rate of irrational medical orders was 1.66%, and the rate of successful intervention was 99.06% (Table 4). After the establishment of the prescription-checking system, the rate of irrational medical orders was decreased, and the success rate of intervention was increased. The differences in medical order qualification rate and intervention rate before and after the implementation of the database were statistically significant (*P* < 0.05 for both; chi-square test).

**Table 4:** Common types of irrational medical orders and the rate of success intervention

Group	Number of medical orders	Number of irrational medical order	Rate of irrational medical orders	Number of interventions	Success rate of intervention
1	709,040	16,488	2.33%	14,162	85.89%
2	687,656	11,446	1.66%	11,338	99.06%

### Practical results of the application of intelligent devices in PIVAS

#### Automatic labeling machine VS Manual

**Fig. 8:**
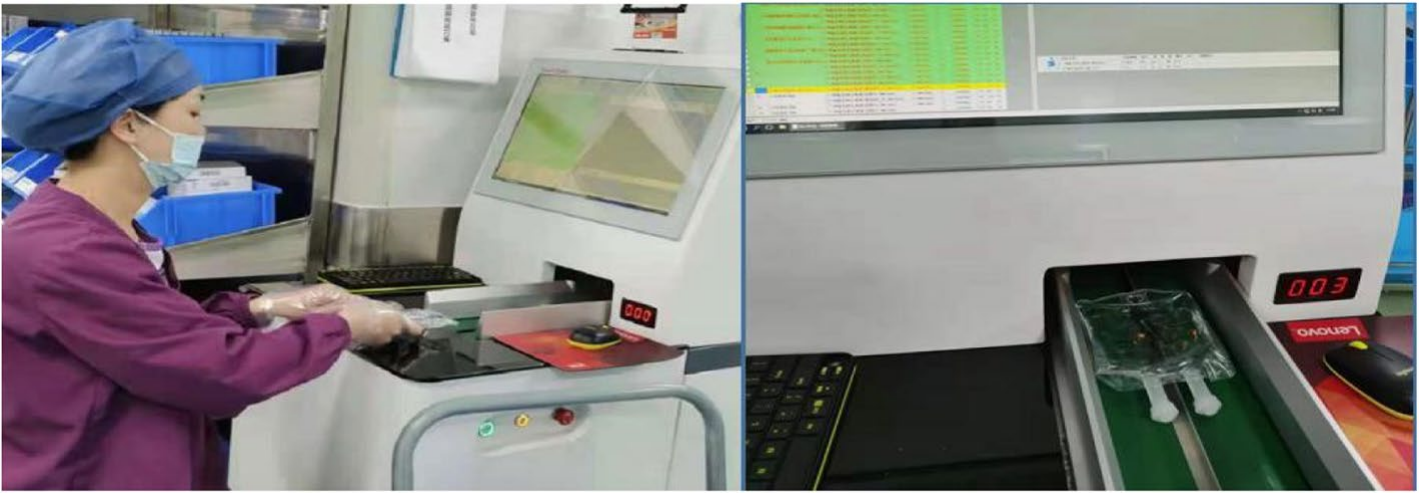
The intelligent labeling machine is used for infusions labelling

Two automatic labeling machines were applied to reduce the risk of labelling errors for the large number and similarity of infusion bags. It is operated by two staff members: one worker placed the infusion bags on the conveyor belt for automatic label printing, another checked the labeled infusion bags. The automatic labeling is suitable for solvents in plastic bags, but not for solvents in glass bottles and solvents with large volumes. To reduce the possible errors, drugs with similar names or different specifications are often labelled by automatic devices and manual respectively. By selecting the button of “machine label printing” in the PIVAS management system, labeling information is automatically distributed to the automatic labeling machine for printing and labelling (Fig. 8). When the label of the infusion bag is completed, the staff would be prompted to replace the infusion bag. The automatic labeling equipment also has the function of image recognition. An alarm is triggered to warn the solvent labeling errors risk, when the solvent information is inconsistent with the infusion lable.

The data for 60 days were collected and the *t*-test was applied to compare efficiency and accuracy between automatic labeling and manual labeling. Automatic labeling was found to be significantly faster and more efficient than manual labeling (*P* < 0.001; Table 5). Within 60 days, manual labeling led to a total of 27 errors, whereas automatic labeling resulted in 7 errors, suggesting that automatic labeling was more accurate than manual labeling.

**Table 5:**
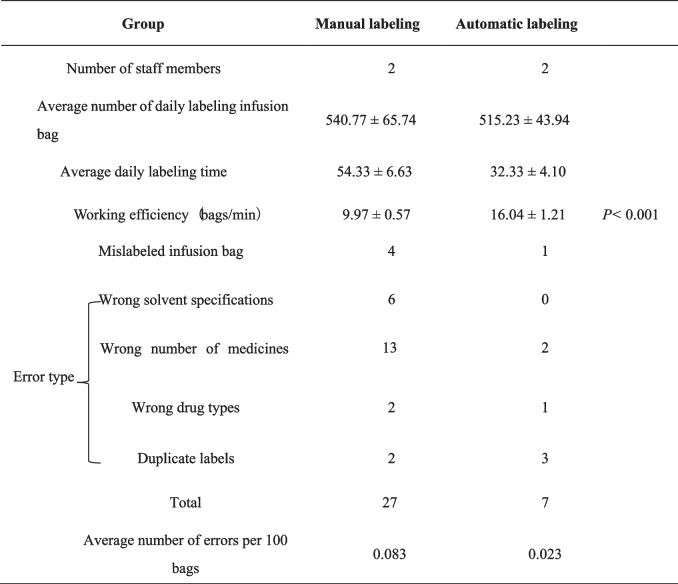
Comparison of efficiency and accuracy between manual and automatic Labeling

#### Intravenous compounding robots VS Manual

**Fig. 9:**
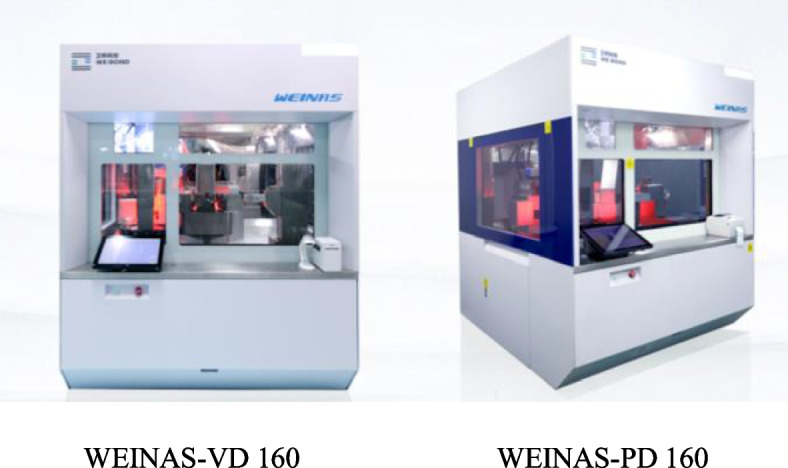
Intelligent intravenous medicine compounding robots

To reduce the risk of occupational exposure to toxic agents and improve the efficiency and accuracy of medicine admixture, two automatic robots are used to prepare the compounding of intravenous chemotherapeutic drugs (Fig. 9). The robots have many advantages, such as robot vision and gravity sensing technology, a powerful database system, advanced sensing and control algorithms, mechanical power (multi-degree-of-freedom mechanical arm and omnidirectional liquid medicine transmission system), the preparation of chemotherapy admixtures was performed automatically.

**Fig. 10:**
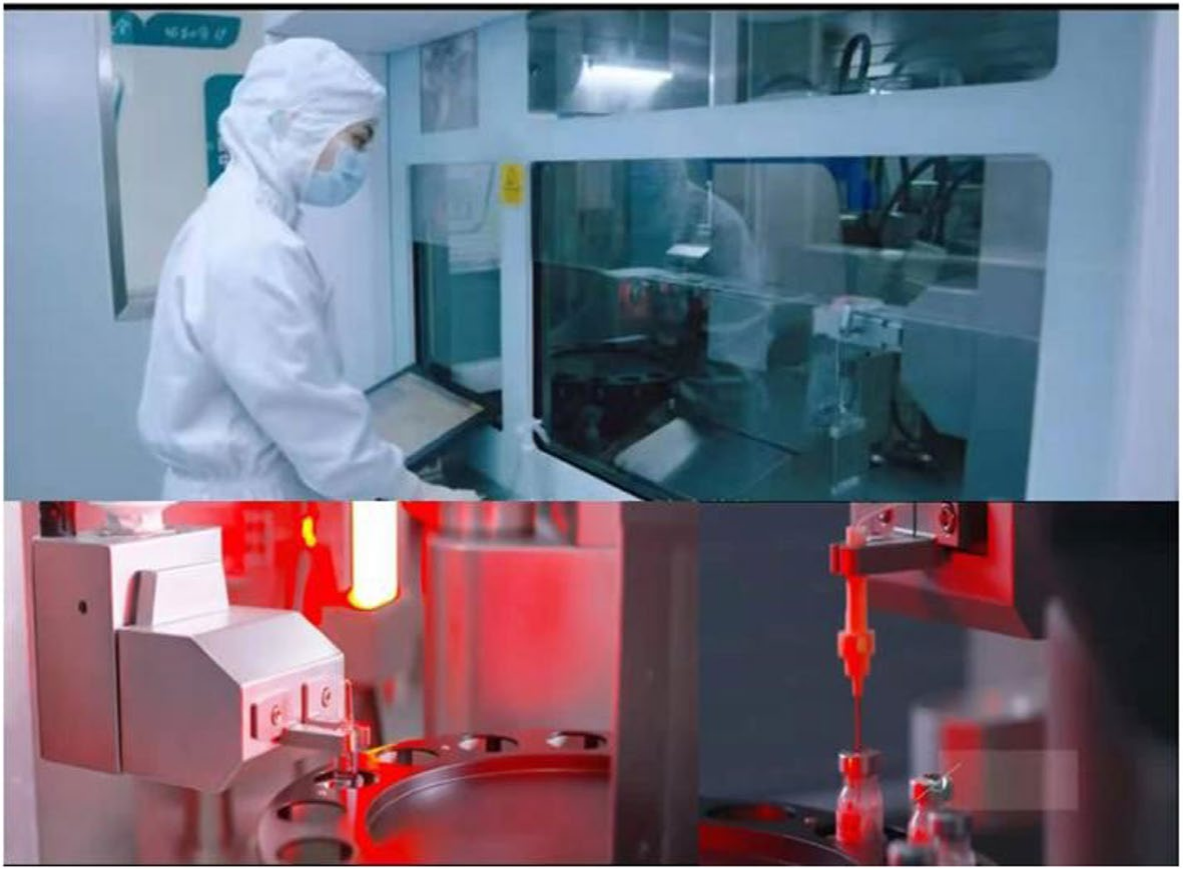
The intelligent intravenous compounding robots are used for cytotoxic drug admixing

Drug characteristics, prescription requirements, machine stability, and drug admixture accuracy may affect the quality of robot-prepared infusion product. By establishing a quality procedure of intravenous compounding robot, the dosing accuracy of the infusion is nearly 95% confirmed by sense, weight, and volume algorithms. We conducted a data demonstration for all cytotoxic drugs in PIVAS by verifying the parameters of the injection volume and dissolution requirements, and only drugs meeting the standards were prepared by the robots. This standard procedure also enabled the intelligent robots to identify and review prescriptions. The process of drug admixture preparation was monitored in real-time. We found that the error in robotic accuracy was within ± 3%. Moreover, robots can be used to prepare diverse drug admixtures, such as non-whole ampoule bottles and mixed dispensing of drugs. Two staff members are responsible for the compounding of cytotoxic drugs by the two robots (Fig. 10).

The accuracy and efficacy of manual and robotic preparation of chemotherapy admixtures was compared. The relevant data was collected within 60 days and the *t*-test was used to determine the differences on accuracy and efficacy between manual and robotic preparation of admixtures. Manual was significantly more efficient than robotic (*P* < 0.001; Table 6). However, automated compounding was more accurate than manual (two drug admixture errors vs. eight drug admixture errors). Therefore, implementation of intelligent admixture robots can reduce the error rate and occupational exposure risk.

**Table 6:**
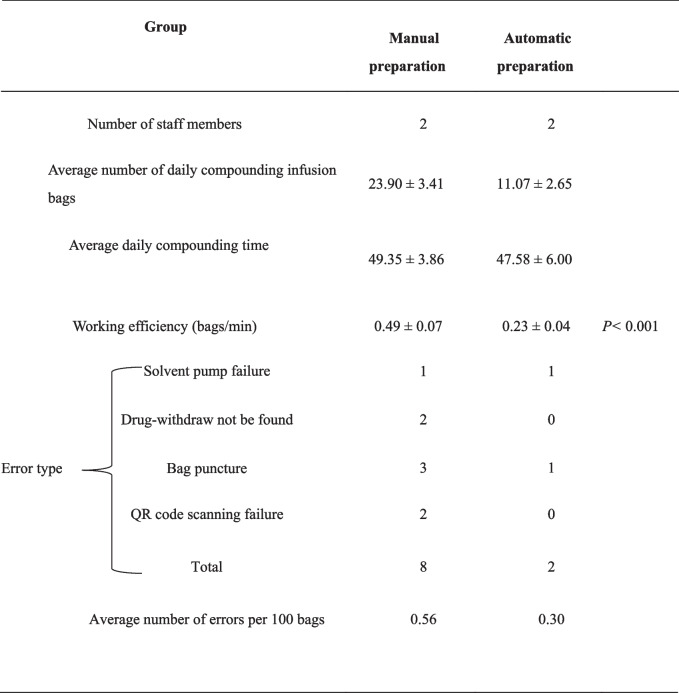
Comparison of efficiency and error rate between intelligent and manual compounding of intravenous chemotherapy

#### Intelligent infusion bag sorting system VS Manual

To minimize the risk of infusion admixtures being sent to the wrong ward and reduce labor costs, an intelligent infusion bag sorting machine was implemented. The intelligent sorting machine can automatically sort infusion bags for 48 wards at most. The number of boxes for the different wards could be configured in the intelligent machine according to the work situation. The machines display the number of sorted infusion bags on the screen while sorting. When the infusion bag has the same information, the machine will voice prompt and put it into the manual waiting area after the alarm, and then the cause of the repeated information will be manually checked out.

**Fig. 11:**
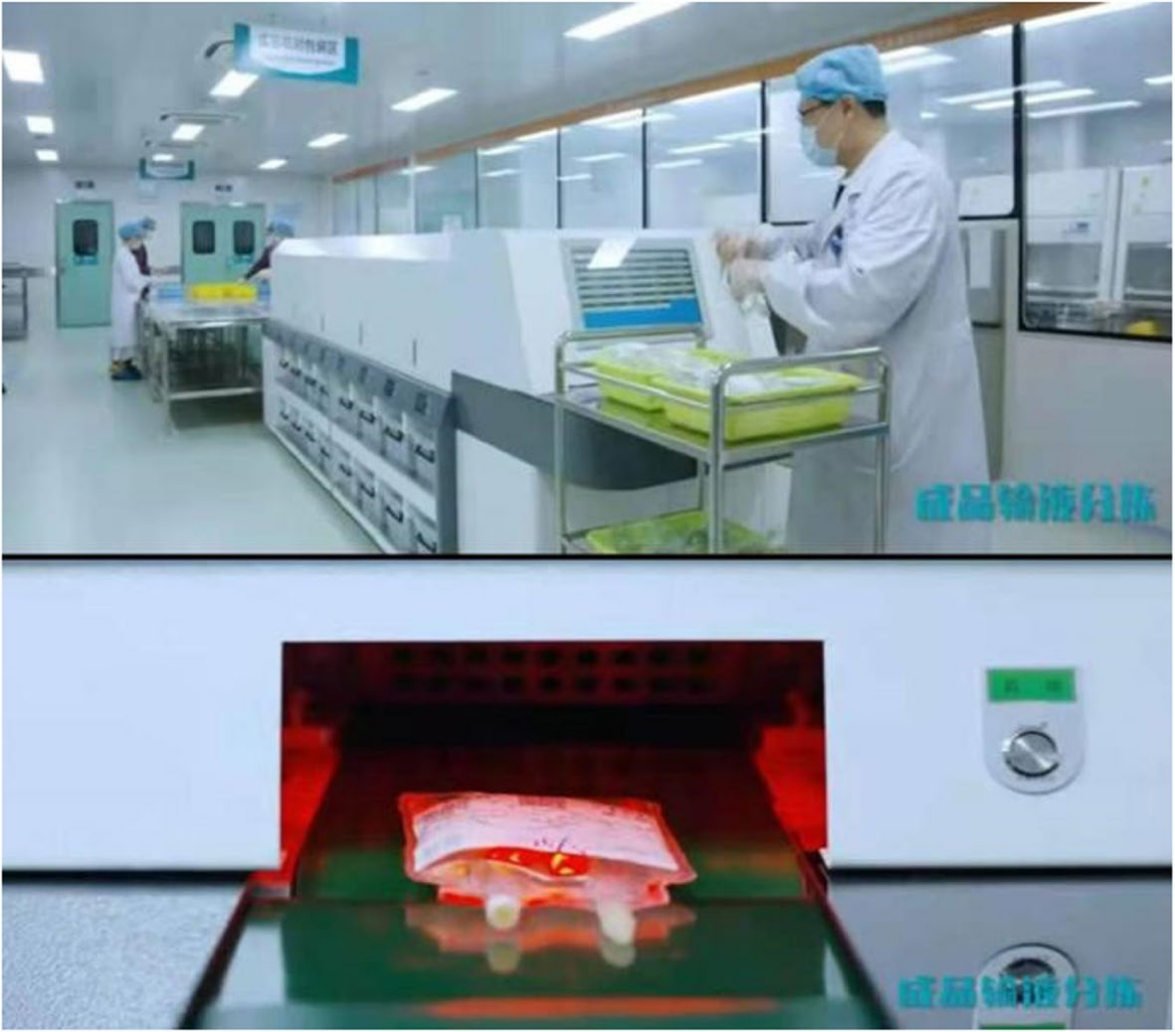
The PIVAS staff operating the intelligent sorting machine

Intelligent bag sorting was dramatically reduced the incidence of sorting errors. However, they occupy a large area, so PIVAS can only introduce two machines at present. They are suitable primarily for small-sized solvents in plastic bags, but not suitable for glass bottle solvent. Each sorting machine in our PIVAS (Fig. 11) operated at an efficiency of 720 bags per hour (each requiring two staff when in use), indicated that the system may not suitable for the first (6000 bags) and second (3000 bags) batch of infusion admixtures, but only can be used for the third batch of infusions (1000 bags). Although the error rate of sorting was much lower than that of manual sorting, but it has a lower sorting efficiency than manual sorting,which could be participated in by more people.In an hour, 20 people could sort out 6,000 bags, but the machine couldn't.Therefore, intelligent sorting system is suitable for PIVAS in small and medium-sized hospitals, but the utilization rate of super-large hospitals may not be high.

#### Intelligent logistics robots VS Manual

After the preparation of intravenous infusions, admixtures are sealed into boxes and delivered to inpatient wards. However, missing infusions and delivery errors may occur. Additionally, logistics delivery personnel has a high risk of potential infection when they go in and out of clinical departments. Therefore, the implemented of logistics robots was very necessary.

**Fig. 12:**
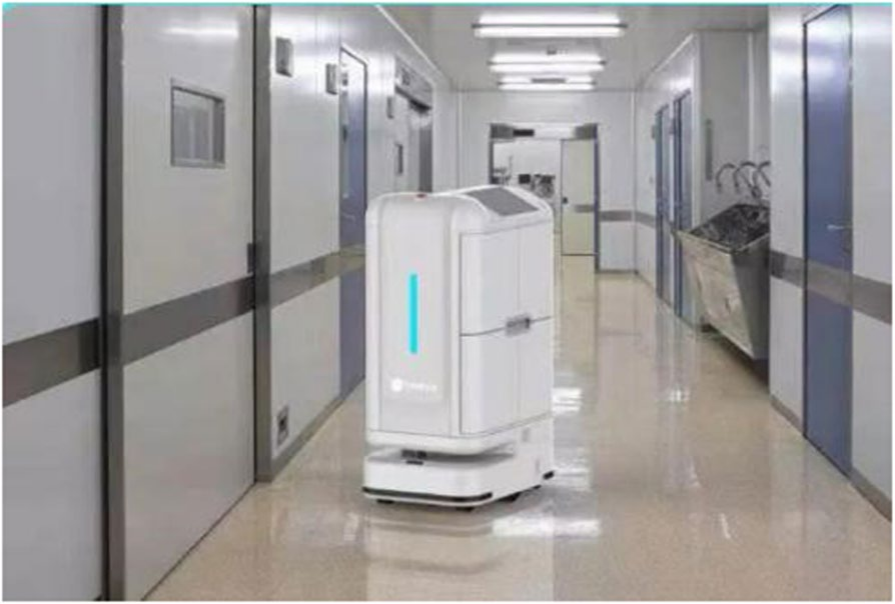
The PIVAS intelligent logistics robots delivering infusions to inpatient wards

The implementation of logistics robots can reduce the requirement for workforce, material resources, and protective equipment. However, robots can only deliver a small number of infusions and require supporting hardware and software for different locations (e.g., passageway, ground floor, and elevator). Due to the late introduction in our hospital, there is no designed special channel for logistics robots, so the flexibility and delivery speed are far inferior to manual delivery.We currently only have two intelligent logistics robots to automatically identify delivery destinations (Fig. 12). At present, under the operation of just one staff member, two robots deliver the prepared infusion bags from PIVAS to five inpatient wards in the same building. In order to save time and ensure the infusion can be delivered to the ward on time, we still mainly rely on manual delivery.

## Discussion

Characteristics of PIVAS make the system ideal for ultra-large tertiary hospitals performing multiple procedures under heavy workload, time pressure and quality control requirements. There are many prescription audits and drug checks every day, and mistakes in any procedure may result in intravenous infusion errors. To minimize the risk of errors, we need to pay attention to each process at all times. Consequently, future work is required to optimize PIVAS procedures and maximize the integration of information-intelligence technologies and the PIVAS procedures management system. Wuhan Union hospital is affiliated with the National Health Commission of China and took the lead in exploring the application of information-intelligence technologies in PIVAS which play a crucial role in increasing work efficiency, reducing the risk of errors, and enhancing occupational protection. Unfortunately, automatic sorting machines and logistics robots have several disadvantages in practical daily work, resulting in low utilization ratio. As a result, we haven't collected enough data to compare their efficiency vs manual. At present, we are also actively communicating with manufacturers and hospitals, aiming at solving the sorting machine and logistics robot problems. We hope that manufacturers can give a reasonable solution to increase the efficiency of the machine. At the same time, when introducing intelligent equipment, it is also hoped that hospital colleagues should combine their own working conditions and fully consider whether intelligent equipment can meet the daily work needs. It is hoped that all intelligent equipment for PIVAS can be further developed in the future, so that the work efficiency of PIVAS can be increased in an all-round way.

## Conclusions

PIVAS offers several advantages in terms of occupational protection and medication safety. However, intravenous infusion can cause adverse reactions at a higher rate than oral administration. Therefore, intravenous infusion safety is critical for treatment outcomes. The application of information-intelligence technologies can improve medication safety. PIVAS can be particularly useful in large tertiary hospitals with a heavy workload, and the larger the scale of PIVAS, the more urgent the demand of intelligent service. Additionally, implementation of intelligent prescription reviewing systems can significantly improve work efficiency and minimize medical order errors, and intelligent devices can improve occupational protection. However, certain limitations need to be addressed to maximize the benefit of PIVAS, including limited compounding speed, low flexibility of intelligent compounding robots and automatic sorting machines, the high costs of intelligent equipment, and huge need for trained personnel.

## Data Availability

The datasets generated and/or analyzed during the current study are not publicly available because they are subject to the Union Hospital, Tongji Medical College, Huazhong University of Science and Technology. However, the data and materials are available from the corresponding author on reasonable request.

## Abbreviations

PIVAS: Pharmacy intravenous admixture services
TPN: Total parenteral nutrition
COVID-19: Coronavirus disease 2019
EMR: Electronic medical record system
HIS: Hospital’s information management system
